# Research Progress on Insulated Gate Bipolar Transistor (IGBT) Modules

**DOI:** 10.3390/mi16020197

**Published:** 2025-02-08

**Authors:** Peisheng Liu, Yaohui Deng

**Affiliations:** 1 Department of Information Technology, Xinglin College Nantong University, Nantong 226236, China; 2 Jiangsu Key Laboratory of Semiconductor Device and Integrated Circuit Design, Packaging and Testing, School of Microelectronics and School of Integrated Circuits, Nantong University, Nantong 226019, China; 13016782818@163.com

With the rapid advancement of social productivity and technological innovation, the insulated gate bipolar transistor (IGBT) has emerged as a cornerstone in modern power electronic devices [1,2,3]. This unique device integrates the benefits of a bipolar junction transistor (BJT) and a metal–oxide–semiconductor field-effect transistor (MOSFET), achieving a remarkable balance between a high impedance, efficient voltage control, and a superior current handling capacity [4,5]. IGBTs are indispensable in a wide array of high-power applications, including wind turbines, high-speed trains, electric vehicles, and maritime propulsion systems [6]. Their ability to operate at high switching speeds with low on-state voltage drops makes them ideal for managing high-efficiency energy conversion in complex electrical systems. As energy demands escalate globally and sustainable technologies gain traction, IGBTs have played a pivotal role in the development of smart grids and high-voltage DC transmission systems, where reliability and efficiency are paramount. Despite their advantages, IGBTs face challenges related to prolonged high-temperature operation, which can result in increased failure rates and reduced reliability [7,8]. In systems with low fault tolerances, such failures may lead to irreversible damage and significant operational risks, highlighting the critical need for ongoing research and innovation in IGBT design, material engineering, and packaging reliability [9,10].

This Special Issue on insulated gate bipolar transistor (IGBT) modules includes 11 selected papers that address pressing challenges in the field. The topics covered span reliability assessment, material innovations, thermal management, fault detection, and performance optimization, providing a comprehensive overview of recent advancements and future directions.

In particular, Yan et al. [Contribution 1] demonstrate the superior thermal and mechanical reliability of nano-silver solder in GaN chip packaging under high-temperature and high-humidity conditions. These findings provide a robust basis for enhancing high-performance GaN chip packaging. Xu et al. [Contribution 2] quantify the effects of void size and location within solder layers on the thermal performance of IGBT modules. Their thermal model offers actionable insights into optimizing soldering processes. Hamieh et al. [Contribution 3] explore the impact of thermal fatigue on grain groove profiles in thin polycrystalline films. Their advanced microscopy techniques reveal strategies to mitigate grain coarsening, thereby improving the thermal stability of power devices.

Hu et al. [Contribution 4] propose a novel active thermal control strategy for IGBT modules, achieving finite-time boundedness to optimize performance under dynamic load conditions. Additionally, they develop an improved thermal equivalent circuit framework for real-time temperature prediction, enhancing the precision of thermal management [Contribution 5]. Furthermore, Hu et al. [Contribution 6] introduce an online detection mechanism for bond wires that have fallen off IGBT modules, providing a proactive fault identification approach that improves device reliability.

Chen et al. [Contribution 7] apply a BiTCN–Attention transfer learning model to achieve high-precision fault prediction for power devices in rod control power cabinets. This approach facilitates proactive maintenance and reduces system failure rates. Qian et al. [Contribution 8] propose a novel trench–gate bipolar transistor design with enhanced short-circuit ruggedness and operational speeds, addressing challenges in high-performance applications. Zhang et al. [Contribution 9] explore gate boost drive technology to significantly enhance the overload capacity in traction converters, a critical improvement for heavy-duty applications. Shieh et al. [Contribution 10] design a high-performance multilevel inverter with a simplified and symmetrical structure, reducing component count and control complexity while maintaining a superior output performance. Sheikhan et al. [Contribution 11] investigate a hybrid power switch combining a silicon IGBT and a silicon-carbide MOSFET. This hybrid switch reduces switching losses and enhances voltage withstand capability, presenting a novel solution for high-voltage applications.

The contributions in this Special Issue significantly advance the understanding and development of IGBT modules, addressing challenges in reliability assessment, thermal management, performance optimization, and the integration of emerging technologies in production processes.

We extend our heartfelt gratitude to all contributing authors and reviewers for their dedication and insightful contributions. We believe that the research presented in this Special Issue will serve as a valuable resource for academics and industry professionals, fostering further advancements in the design, reliability, and application of IGBT modules.
